# Tumor on the Thigh, Affecting the Arterial System

**Published:** 1845-09

**Authors:** E. L. Dudley


*Tumor on the Thigh; affecting the Arterial System.* Reported by E. L. Dudley, M. D.—In the fall of 1838, a black man, of middle age, was sent to Professor Dudley, with a large tumor, situated upon the inner half of the thigh. The patient did not suffer in the limb, and the tumor might be freely handled without any inconvenience to him. There was nothing in the appearance or position of the tumor to justify the belief that it was of a malignant character, and yet the pulse of the patient, even after he had undergone a course of treatment for two months, was constantly at 140. The operation was finally performed, and a large tumor extracted, which involved three or four inches of sartorious muscle. It was remarkably heavy, and the greater portion of the interior converted into bone. In twenty-four hours after the removal of the tumor the pulse of the patient fell to ninety in the minute, and in a few days to the healthy standard.

How did the presence of this tumor upon the thigh accelerate the action of the heart so remarkably? The patient recovered rapidly without a single unfavorable symptom, and was, two years after the operation, one of the most active men upon his master's farm. If the tumor had been inflamed or the patient unusually irritated under nervous impressions, we might conjecture with some plausibility the cause of this unusual phenomenon. The fact that it was situated immediately over the femoral artery, and admitting that it might have pressed upon the vessel, (which it could not have done, in consequence of its superficial position, to an injurious extent), throws no light upon the case. The general health of the patient seemed to be good from the first, and the rapidity with which the wound healed proved that he was in perfect health at the time when the operation was performed.—*Western Lancet.*

